# Towards a liquid healthcare: primary care organisational and management strategies during the COVID-19 pandemic - a qualitative study

**DOI:** 10.1186/s12913-022-07855-0

**Published:** 2022-05-17

**Authors:** Montserrat Pulido-Fuentes, Luisa Abad González, Isaac Aranda Reneo, Carmen Cipriano-Crespo, Juan Antonio Flores-Martos, Ana Palmar Santos

**Affiliations:** 1 Faculty of Health Sciences, University of Castilla-La Mancha, Avenida Real Fábrica de Sedas s/n, 45600 Talavera de la Reina, Toledo Spain; 2 Faculty of Education Sciences and Humanities, University of Castilla -La Mancha, 16071 Cuenca, Spain; 3 Faculty of Social Sciences, University of Castilla -La Mancha, 45600 Talavera de la Reina, Toledo Spain; 4 Faculty of Medicine, Autonomous University of Madrid, Calle Arzobispo Morcillo n° 4, 28029 Madrid, Spain

**Keywords:** COVID-19, nursing, pandemic, primary healthcare, qualitative study, management

## Abstract

**Background:**

The COVID-19 pandemic has changed the organisational and management strategies of healthcare institutions such as primary care centres. Organisational culture as well as leadership style are key issues for the success of these institutions. Due to the multidimensional nature of identity processes, it is necessary to explore the changes experienced by health professionals from these perspectives. This study explores health professionals’ organisational and management strategies in primary care settings during the COVID-19 pandemic.

**Design:**

Qualitative, exploratory study based on the analysis of participants’ accounts within a hermeneutic phenomenologicaly approach.

**Methods:**

Research was conducted in primary care settings in two neighbouring Spanish healthcare regions. The sample included participants with different demographics (gender, age), professional roles (practice managers, general practitioners, paediatricians), employment status (permanent, temporary, zero-hours), and years of experience (under or over ten years’ experience). Data were collected between July and December 2020 through focus groups and in-depth, semi-structured individual interviews.

**Results:**

A total of 53 primary care workers participated in the study, of which 38 were individually interviewed and 15 participated in three focus groups. Of these, 78.4% were healthcare professionals, 49% were female nurses, and 70.5% had more than 10 years of work experience in primary care. Two main themes emerged: “liquid” healthcare and “the best healthcare system in the world”. During the first wave of the COVID-19 pandemic, new, more fluid organisational and management models were implemented in primary care settings, which have remained in place since. Primary care workers’ perceived a lack of appreciation and inclusion in decision-making that risked their alienation and disengagement.

**Conclusion:**

Primary care workers’ professional identity became gradually blurred due to shifting perceptions of their professional roles in a context of increasing improvisation and flexible working practices. This affected their professional performance.

**Trial Registration:**

The study was approved by the Clinical Research Ethical Committee of the Talavera de la Reina Integrated Management Area (CEIm del AGI de Talavera de la Reina in Spain, Hospital Nuestra Señora del Prado, ref: 23/2020).

## Background

The World Health Organisation (WHO) has stressed the role of primary care as the most efficient and effective strategy to improve the health of populations [1]. It has also emphasised the importance of ensuring the capacity of primary care settings to continue delivering essential services during the pandemic, as well as monitoring self-isolating cases [2, 3]. Managing the COVID-19 pandemic has proved an unprecedented challenge to healthcare systems globally, affecting and delaying planned interventions [4, 5] and forcing a reorganisation of existing, well-established assistive care models [6]. The pandemic scenario has placed a greater workload on healthcare systems, already weakened as a result of funding cuts introduced in Europe countries after the financial crisis [7]. This fragility hindered efforts to direct and allocate existing resources effectively.

Primary care centres are the cornerstone of the Spanish healthcare system, and the first point of access from which patients are filtered and referred to other care levels – acting as “gatekeepers”, in a similar way to general practitioners in the British National Health Service [8]. Primary care is essentially delivered by public providers and is funded mainly through general taxation.. However, challenges posed by the COVID-19 pandemic strained an already vulnerable healthcare system, contributing to its increasing fragility [9, 10]. Problems accessing healthcare systems – particularly primary care – and delivering high-quality patient care during the COVID-19 pandemic have increased globally [11, 12].

Countries worldwide have significantly transformed their healthcare systems to better respond to this global crisis [6]. Since the 1950s, corporate or organisational culture has been turned into a mechanism to increase efficiency and management control within organisations [13, 14]. Organisational culture, discussed by numerous authors, exerts a considerable influence, especially in areas such as performance, morale, employee commitment and loyalty, employee attitudes and motivation and efforts to attract and retain talented individuals [15] as well as on professional identity [16, 17]. Organisational culture plays a central role in determining strategies, goals, practices, and results in working environments – a positive organisational culture is associated with higher job satisfaction and commitment among workers and fewer adverse events [13]. Organisational culture affects resource management, intervention planning, and professional roles within healthcare systems. Another key issue for the success of organisations is leadership style, which is fundamental to the success of organisations [18, 19]. Without strategic and effective leadership, it is difficult for organisational members to maintain profitability, productivity and competitive advantage [20, 21].

Primary care organisational structures must be reassessed to ensure an adequate response to the current, and future, pandemics – for which purpose a more dynamic model is necessary. The main factors underpinning a strong, efficient corporate culture are the alignment of workers with the organisation’s goals, management style, organisational practices, contracting policies, professional development, and workers’ rewards and rights [14], as well as leadership style and job satisfaction [15, 22]. Healthcare workers are an essential component of the institutions in which they conduct their professional practice– which they are defined by and help construct their work identity [23, 24] promoting the adoption of specific organisational strategies [25]. Therefore, institutions are shaped by their workforce’s cultural and political beliefs, in addition to external factors such as national policies [26]. Institutions do not exist in isolation, but in a particular macro-social context [25, 27, 28] . At the same time, institutions justify their existence with specific aims and goals, which they seek their members to identify with. However, in the present era of liquid modernity [29], with its fluid, unpredictable social conditions, the value of social cohesion as an integrating factor seems to be in decline.

In this context, leadership style is a critical variable affecting workers’ behaviour within healthcare organisations [30, 31]. It is also a predictive factor of professional performance [32] and the patients’ perception of the quality of care received [31]. Therefore, it is essential to identify efficient strategies to respond to the current pandemic while maintaining high-quality, resilient healthcare systems [33, 34]. These strategies could help shape future evidence-informed policy decisions regarding the optimal organisation and management of primary care resources. We aimed to explore how the management and organisational strategies were applied in Spanish primary care centres during the first wave of the COVID-19 pandemic from the point of view of healthcare workers.

## Methods

### Aims

The aim of this study is to explore the management, organisational strategies, and response capacity of primary healthcare centres in two different Spanish health regions to cope with and adapt to the pressure of the COVID-19 pandemic, from the viewpoint of frontline healthcare workers.

### Design

We designed a qualitative, exploratory study [35, 36] under a phenomenological paradigm that follows hermeneutic approach. It focuses on the relationship between an individual and their life trajectory and culture, which influences the conscious way in which they experience a phenomenon. Hermeneutic phenomenology must go beyond describing a phenomenon – it must interpret it, exploring the person's consciousness and understanding how they give meaning to aspects of their life [37] to explore personal perceptions of the phenomenon studied. An inductive thematic analysis of semi-structured interviews [38] and focus groups [39, 40] was conducted. This approach allowed us to explore the significance that participants attributed to their personal experiences and perceptions of institutional organisation during the COVID-19 pandemic.

### Sample/Participants

Study subjects were primary care workers of the Castilla-La Mancha Healthcare Service (SESCAM) and the Madrid Region Healthcare Service (SERMAS). These regions were selected for the study due to the similarity of their profiles in terms of incidence during the first surge of the COVID-19 pandemic, management of healthcare resources, restrictions in access to the healthcare system, and economic and socio-demographic factors [41, 42]. Purposive sampling was used to increase diversity in participants and results. To maximise variability in the experiences collected, sampling was based on the following criteria: demographic profiles (gender and age), professional roles (practice managers, general practitioners, pediatricians), employment status (permanent, temporary, zero-hours), and years of experience (under or over ten years of experience) ensuring there were at least two subjects each with different profiles. We also considered whether these workers had dependent family members (Table 1). Invitations to participate in the study were sent via institutional e-mails to more than 600 healthcare professionals working in the regional healthcare system where the study was conducted (convenience sampling). Participants answered questions regarding their health status, job characteristics, socio-demographic profiles, and the Burnout Clinical Subtype Questionnaire (BCSQ-36). Once the survey was completed, participants were asked whether they wanted to join the qualitative research phase, either via individual interviews or focus group discussions. A total of 677 invitations were sent, of which only 37% resulted in acceptance to join the project. From this 37% (*n* = 252), only 22% (*n* = 56) agreed to participate in the qualitative phase. We also used snowball sampling, with initial participants identifying additional subjects among their contacts. Those who expressed their willingness to participate in an individual interview or focus group and fitted the target profile were sent an e-mail with information on the study’s aims, anonymisation, and personal data processing procedures. The research team responded to queries from the participants via e-mail or telephone calls.

**Table 1:** Characteristics of participants included in the study

	Number of Participants
Male/Female	16/37
Rural/Urban	21/32
Over/Under 10 Years’ Experience	38/15
With/Without Dependent Family Members	27/26
Nurses	26
General Practitioners	2
Nursing Managers	5
Practice Managers	4
Nursing Aides	3
Emergency Technicians	2
Social Workers	3
Physiotherapists	3
Administrative Staff	2
Midwives	2
Cleaners	1

### Data Collection

Data were collected through in-depth, semi-structured interviews [38, 43] and focus groups [39, 40], which were conducted between July and December 2020.

Most individual interviews were completed in-person, in venues selected by the participants themselves, although some took place via telephone or video calls. A total of 38 individual interviews were conducted, with durations of between 45–70 minutes each. Interview guides (Table 2) were developed by the same researchers who conducted the interviews, all of whom were experienced in qualitative research.

**Table 2:** Interview guide

Subject Areas	Questions
Working in a primary care setting	• Describe an average working day before and during the COVID-19 pandemic. • Compared to the situation before the pandemic, how has the health crisis affected your working environment and professional performance? • How have the work management practices and care provision planning been affected? What resources have been used? What resources have been made available? • What communication channels have been used regarding information, training, protocols, etc.? How have you received information? What was its quality?
Working as part of a team	• Describe how the relationships with the rest of the team have been. Has anything changed during this time? • What did the team members say about what was going on? • Have there been any cases of infection within the team? What was your experience of this? Is there anything you would like to mention regarding the team?
Healthcare provision	• Compared to the situation before the pandemic, how has the health crisis affected users, patients, families, the community, and primary care in general? • What have been the main types of consultations/demands for primary care services during the lockdown period? What has been the impact on care delivery, particularly for the chronically ill, dependent population, and vulnerable groups?
Wrap-up issues	• Considering the situation pre- and post-COVID, what kinds of relationships should be established between primary care and community resources, other professional institutions, and other care levels? • What roles should primary care play in pandemics, health crises, or emergencies such as the current one? Where should efforts be focused to control this pandemic and prevent new outbreaks? • What would you change regarding the provisions adopted in primary care during this crisis?

Focus groups were conducted in rooms that were comfortable and guaranteed confidentiality while adhering to COVID-19 health guidelines, allowing healthcare workers to engage in a candid discussion of their experiences. A total of 15 professionals took part in three focus groups, with durations of around 120 minutes each. Focus groups followed a discussion guide (Table 3) that the research team prepared after a preliminary analysis of questionnaires and interviews. They were attended by professionals who indicated their willingness to participate in the qualitative research stage. Discussions were led by different research team members, with help from another researcher in their preparation and planning.

**Table 3:** Focus group discussion guide

Treatment received by primary healthcare from institutions and public opinion. Feelings that this treatment elicited.	
Experiences of primary care emergency services being close to collapse during the first and second waves of COVID-19.	
Institutional organisation of services during the pandemic. Possible ways of improving it.	
Factors to improve and optimise primary care resources. Who is responsible for resource allocation and optimisation?	
Structure of work teams. How team-based work was carried out.	
Existence of physical and/or verbal aggression. Situation before the pandemic. Changes after the pandemic.	
Meaning of teleconsultation for healthcare professionals and users. Teleconsultation in rural areas and for older adults.	
Team-based work environment before and during the pandemic. Out-of-the-ordinary situations.	
Personal cost of professional performance.	
Strategies and dynamics to manage conflict within teams.	
Possible actions to improve internal organisation in primary care.	

Interviews and focus groups were audio-recorded [38], and a fieldwork diary was used to record contextual issues and the researchers’ observations and thoughts [44, 45]. Only members of the research team had access to data collected throughout the study. None of the researchers involved in data collection had any work relationship with the study subjects.

### Data Analysis

Interviews and focus group discussions were transcribed verbatim. All members of the research team read the transcriptions to familiarise themselves with the accounts. A thematic map was prepared with themes and subthemes, illustrated with relevant quotations from the participants’ accounts. Following an inductive thematic analysis approach [46], a phenomenological perspective was used for a more descriptive analysis of the participants’ responses, identifying significant meaning units. Texts were compared and analysed to identify emerging categories [47]. During a second stage, a more in-depth analysis was performed to identify common categories.

This procedure was repeated by each member of the research team for each interview, focus group discussion, and field notes and observations – all of which were independently coded. The results of their individual analyses were discussed in team meetings, where significance units were agreed upon through consensus between all researchers. Each participant was allocated an alphanumeric code for data logging, category creation, and as a reference for literal quotations from the participants’ accounts.

All participants were offered the opportunity to review the audio or written records as well as the subsequent analysis to confirm the interpretation of their narratives by the researchers

### Rigour

The study followed the COREQ guidelines for reporting qualitative research [48], as well as Lincoln and Guba’s quality criteria for qualitative research [49] (Table 4).

**Table 4:** Data reliability criteria

Criteria	Procedures and techniques to establish quality criteria
Credibility: confidence in the trustworthiness of the researchers’ findings and interpretations	• Analyst triangulation: each account was individually analysed before these results were compared in team discussions, where common categories were identified and agreed upon. • Participant triangulation: the analysis included participants with different profiles and viewpoints. • Triangulation across different data sources and collection methods – semi-structured interviews, focus groups, and field notes. • Participants’ validation: participants were offered the opportunity to review their audio recordings and corresponding transcriptions, as well as the subsequent analyses.
Transferability: observations are representative	• Information is provided on data collection and analysis procedures, researchers’ profiles, participants’ profiles, and context.
Dependability: the study is consistent and data are stable	• Different triangulation techniques are used. • Experienced researchers reviewed the quality of the research procedure.
Confirmability or neutrality: to what extent results were shaped by participants and not research bias	• Reflexive examination of researchers, through field diaries and triangulation of methods, researchers, and analyses. • Description and triangulation of participants’ profiles and their selection process. • Use of mechanisms for recording and analysing transcriptions.

## Findings

A total of 53 primary care workers participated in the study, of which 38 were individually interviewed and 15 participated in three different focus groups according to the saturation criteria of the subject areas. The majority were healthcare professionals (78.4%), female (72.5%), with over 10 years’ experience in primary care (70.5%).

**Fig. 1:**
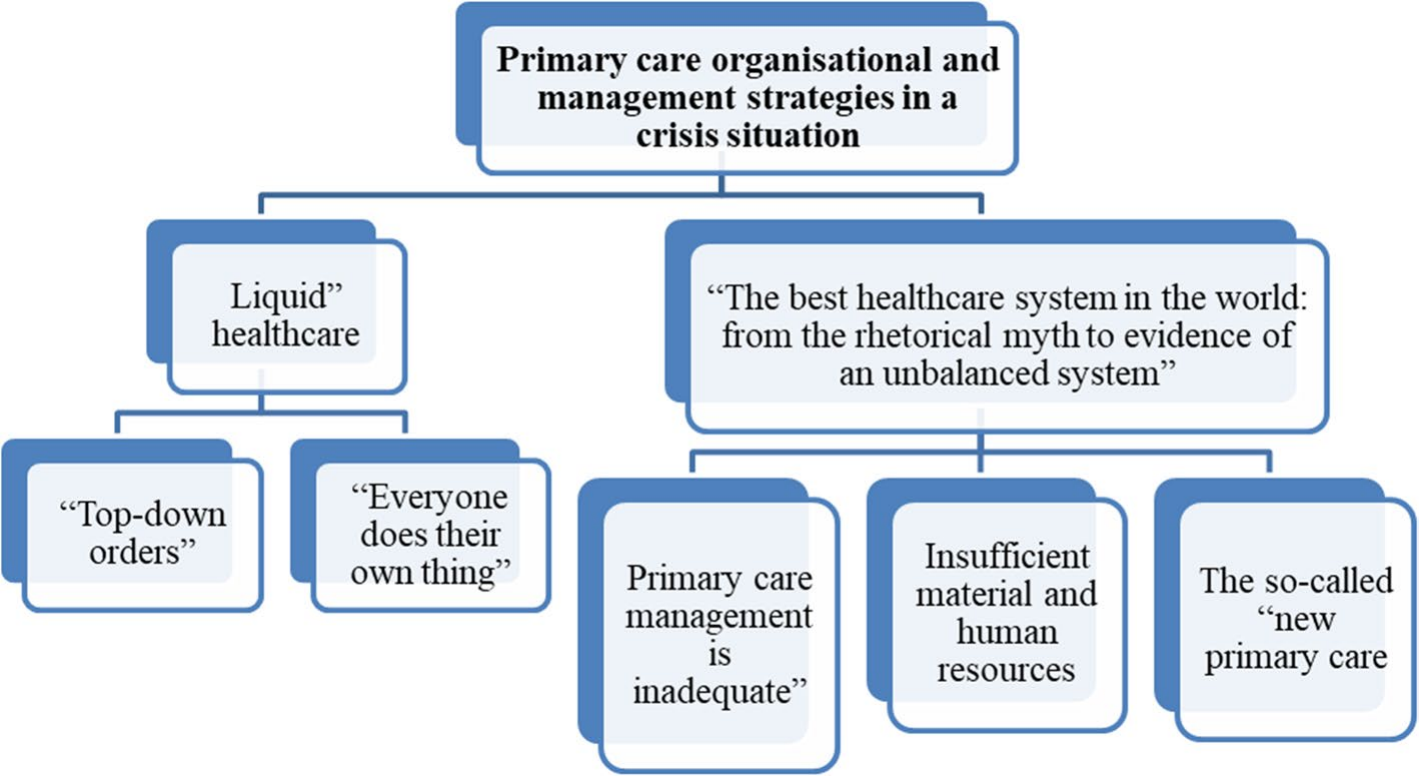
Coding tree for thematic analysis

Two common categories emerged from the analysis of the data collected through in-depth interviews and focus groups in the two healthcare regions where the study was conducted. These describe the impact of the COVID-19 pandemic on the Spanish National Healthcare System from an organisational culture perspective. The results are shown in Fig. 1

### “Liquid” healthcare

Our participants perceived that their working environments during the COVID-19 pandemic were increasingly imbalanced and conflicted – stressing the contrast between the day-to-day reality they experienced at work and the institutional protocols and guidelines defined by healthcare authorities. Their feelings of having been left to cope with whatever resources they already had, in a context of “liquid modernity”, suggested the following categories:

### “Everyone does their own thing”

Healthcare professionals’ personal experience was based on a departure from their usual work routines – with the abrupt implementation of highly fluid organisational strategies. Different actors at different management levels – the Spanish Ministry of Health, regional healthcare authorities, and primary care centres – used varying criteria to define their action plans and protocols. The concatenation of sometimes contradictory guidelines was counterproductive and inadequate for solving the problems faced by healthcare professionals on their day-to-day practice. Our informants described these clashing criteria as “improvised”, “incoherent”, “uncertain”, “lacking coordination”, and “disorganised”. Moreover, their situation did not improve once the first wave of COVID-19 was over.

The lack of clear guidelines forced healthcare professionals to take responsibility for decision-making – which, in turn, caused anxiety and feelings of being abandoned, exposed, and unprotected. Each primary care setting had to self-organise and make their own decisions independently. Institutional, top-down decisions were not aligned with their real, day-to-day experiences and needs. The general perception was that healthcare authorities had been unable to respond effectively to the crisis. As a result, primary care workers had to become responsible for managing the response to the pandemic:

*(...) it caught us with a management model that was not good, because there was uncertainty – nobody defined clear criteria and you had to improvise as you went along, because at the end of the day you are the one dealing with the patient, so you are the one improvising – on a clinical level we have improved, now we know what to do, but as for management it leaves much to be desired, well yes, of course – working conditions were inadequate because nobody sorted it out – I had to make decisions because nobody told us what to do and it had to be done, and by the time the regional healthcare authorities sent their guidelines we were already there. We would have preferred more horizontality, more sharing...* (UM-23, medical coordinator)

According to our participants, the lack of a horizontal approach to decision-making and the top-down communications between different primary care levels complicated their adaptation to the new situation. Paradoxically, the increasing number of team meetings conveyed a subjective perception of chaos and disorganisation.

*What I cannot understand is how we got to this point, now, with no organisation whatsoever – because the organisation, I’ll say it again, from my point of view – it was chaotic* (nurse, focus group 2).

### “Top-down orders”

From the beginning of the pandemic until December 2020, when our fieldwork ended, organisational communication was perceived as inadequate to efficiently manage a public health crisis unparalleled in the last century. There had been a trend towards hypercommunication through a variety of channels *–* mostly e-mails, telephone calls, and even informal messaging applications such as WhatsApp – which was clearly inappropriate for interprofessional communication within the healthcare system. The use of personal communication channels instead of the official ones, and the messages failing to provide clearly written, agreed-upon guidelines, increased the unease and uncertainty felt among primary care workers.

*(...) everything was over the telephone and the solutions did not make any sense* (nurse, focus group 1)

The lack of response to their demands and the perception of a sustained “administrative silence” filled primary care workers with indignation. It was also counterproductive for maintaining motivation in an already complicated context.

*(...) what you felt sometimes was anger – you got annoyed because you did not get an answer to your demands* (RN-15, nurse)

In addition, there was a subjective perception among primary care workers that they were not being treated the same as other healthcare professionals. This was partly because of poor communication between different care levels and the dysfunctional healthcare system organisation, as suggested by the Primary Care Physicians’ Forum [50]. Healthcare authorities never sought their opinion, and decision-making was not consensual.

*I would like them to listen more to those of us that are on the frontline – I would really like that, because they do not listen to us, and that is the problem – perhaps if they listened a bit more we would feel better* (UN-2, nursing manager)

*Communication with regional authorities... It has remained more or less as it was. It was always poor – this should make us realise that there is a lot that needs to be changed and improved – for instance, communication with regional healthcare authorities* (RN-24, nursing manager)

### The best healthcare system in the world: from the rhetorical myth to evidence of an unbalanced system

Parties across the political spectrum have touted, over the last decades, the enduring myth of the Spanish National Health System. Electoral pressures have made politicians exaggerate its successes and obfuscate, shifting attention from real problems in what has always been promoted as “the best healthcare system in the world”. In practice, these arguments have masked the consequences of years of far-reaching, repeated pseudo-privatisation policies and dismantling of public healthcare services. Structurally, there is a general lack of awareness and understanding of the role of primary care, which has been magnified during the current pandemic.

*The problem is that regional healthcare authorities do not know how primary care works, they have no idea – even more so in this situation. And I think that, since they know nothing, they should delegate and seek the advice of those who are working in the sector – those are the people who can inform policies and guidelines for primary care, and they would be more useful than the ones in place now* (UAUX9, nursing aide).

### Primary care management is inadequate

The perception that there is a lack of adequate leadership in primary care has been a constant in our participants’ accounts. This perception has been reinforced in the increasingly complex working environments required to deal with the challenges created by COVID-19. The assessment of primary care management strategies from the viewpoint of institutional analysis is revealing.

*(...) we have been a bit adrift – primary care management is inadequate, we need it to change – we professionals try to make things work differently, to have that – to leave uncertainty behind and do things better, because we have the ideas* (UN-12, nursing manager)

Inadequate leadership led some practice managers to step down from their roles. Other professionals applied for early retirement or were considering leaving their jobs after repeatedly denouncing their poor working conditions. During our study, it was observed that several primary care centres lacked medical and nursing managers. Although in some cases this situation pre-dated the pandemic, during its first few months it deteriorated further.

*Nearly twenty years on a temporary contract, almost five as a nursing manager, and when the pandemic hit we achieved the impossible, managing the practice – because nobody wanted to be in charge of medical coordination – and out of the blue, one day I was fired – with my family situation – the constant mistreatment...* (UN-5, nurse).

As a collaborative exercise, our participants described a double situation of subordination: first, as workers with no participation in decision-making, with top-down orders dictated from higher management spheres; and besides that, they received different treatment from colleagues in specialist care.

### The so-called “new primary care”

Working conditions and the work environment are critical for understanding the deficiencies of the primary care model in the two healthcare areas examined in this study. The process of adaptation during the critical first wave of the COVID-19 pandemic brought to the fore renewed calls for a reorganisation of the primary care management model. Some of our participants defined this so-called “new primary care” as one in which patients were assessed remotely, implementing new care delivery strategies. Some professionals were adamant that *“nothing will be the same”* (UN-14, nurse).

For some of them this novel situation was more “relaxed” *–* compared with the previous, overburdened primary care, which was not delivering high-quality patient care. However, this perception was not widely shared *–* other participants described a situation of uncertainty with which they only coped by assuming individual responsibilities.

*(...) not seeing so many people gives you a break, you do not see them – but telephone visits are chaotic, patients do not feel relieved, for people it is disastrous* (UAD-6, administrative staff).

Healthcare workers with extensive professional experience, including in hospital settings, describe a scenario with a predominance of highly experienced staff, at the end of their long careers.

*Yes, yes it is going to change... Regional healthcare authorities must organise their workers differently, too many professionals are high risk – we were the ones at risk, most of us over 55 years old – frontline workers cannot be a high-risk population – we need to rethink organisational strategies so we work better and with less risk* (UN14, nurse).

These professionals demand a new primary care model that recognises the central role of healthcare professionals and the importance of the work they do, giving them back a sense of dignity and self-respect, and resourcing the healthcare system adequately.

### Insufficient material and human resources

Our participants pointed out that the COVID-19 pandemic has magnified structural deficiencies that pre-dated this global health crisis. This affected the healthcare professionals’ working conditions, hindering their capacity to respond to the crisis and putting both patients and workers at risk. For epidemiological reasons, primary care had to radically transform its traditional care delivery model *–* based on direct contact with patients and their close family *–* and implement a virtual patient care model. However, this new model caused conflict among primary care workers and their patients. It highlighted how under-resourced primary care was and the public’s resistance to changes in the traditional, personalised patient care model.

Our qualitative study provided an insight into personal narratives describing the dynamics and the logic underpinning the attitudes of certain population groups, i.e., older, rural adults.

*(...) many people do not have access to a telephone, and they are very, very old, and they still operate under the conviction that if they phone you, they have to pay – so they prefer to come down here, because it is faster and they do not have to spend any money – some of them cannot read, they cannot use a phone, and it was difficult to stop them coming here – they were like “No, I am just coming down to ask you if I could be seen”* (nurse, focus group 2).

In addition, physical facilities in some primary care centres were old and inadequate, making it difficult to adhere to safety regulations essential in a pandemic context. Primary care settings in ancient buildings, with large communal areas but small consultation rooms, complicated the division between “clean” and “contaminated” spaces required to examine patients with respiratory pathologies and/or suspected COVID-19 cases.

*Primary care centres are in a dire state, some of them have never been renovated* (nurse, focus group 2).

Regarding the allocation of human and material resources, the perception re-emerged that primary care and its workforce were not a priority within the Spanish healthcare system. Among other issues, this was related to the problem of overwork, with increased numbers of shifts.

*(...) the feeling is that nobody cares – it is like, you go in and that’s it, whatever will happen will just happen and that’s all, come on – and on, and on, another day, doubling shifts, that’s it, who cares. It’s like this colleague who took medical leave early on because he developed symptoms, and he was away for two and a half months and another colleague took his shifts – nobody ever phoned him to ask how is it going, how are you doing* (RT-3, emergency technician).

Professionals adapted to this novel situation but mentioned the increasing flexibility required from them as the months went by. Careful resource management during the most challenging stages, to alleviate the lack of resources, was crucial.

*(...) I am not the same as I used to be, I am up for anything, I try to help, that is my role, to help as much as I can – if I have to give a hand in physio I do, if I have to help administrative staff I hand paperwork over, if I have to pass on a document I do it – so that the queue there does not reach …* (UAE-7, nursing aide).

*(...) they must think we are elastic chewing gum – the stress is not because of the virus, it is because of the situation – because I do not have enough people, because I cannot do this* (UM-23, medical coordinator).

## Discussion

The revalorisation of the national government, institutions, and management units during the COVID-19 pandemic has resulted in a fragile situation. Their responses to the primary care organisational and management needs emerging during the pandemic were ambivalent and non-specific. Some author suggest this crisis only exposed pre-existing weakness of the public healthcare system, caused by years of austerity policies [10, 51, 52] – whose impact was particularly felt on primary care services [43]. The result of this was a lack of contingency preparedness to tackle a health emergency such as the COVID-19 pandemic, despite the wide range of logistic resources that could have been used to reinforce public healthcare infrastructures.

In a context of chaotic management frontline healthcare workers had to find alternatives. Their prioritisation of individual approaches rather than teamwork during the second wave of the pandemic, when our study was conducted, could be queried – considering the clear advantages of interprofessional collaboration [16, 53]. The communication and attention levels between primary care workers and healthcare authorities were neither fluid nor effective.

Primary care workers did not feel listened to by healthcare authorities. Instead, they felt relegated to a subordinate position, following top-down instructions that did not seek their approval and sometimes conflicted with their day-to-day experiences. As a result, healthcare workers seemly lost their autonomy as individuals and their ability to participate actively in the productive process, which led to their increased indifference. Besides, this attitude was normalised, reducing the healthcare workers’ sense of responsibility for their role in maintaining the system’s health and diluting their professional identity. This situation neglected a fundamental aspect of efficient communication within healthcare organisations [54]: that the existence of fluid, two-way communication channels can foster intentionally collaborative practices and teamwork, critical for performing complex tasks during challenging times such as the present pandemic. These practices have been associated with fewer clinical errors [55] and better outcomes for patients and healthcare systems [53] – positively affecting work dynamics and limiting stress factors among co-workers [56, 57]

As Bleakley [58] suggested, a more fluid dialogue at all levels within a group or institution can help develop the moral values that underpin and strengthen professional practices – with all individuals collaborating in a joint endeavour to achieve a common goal in what is known as joint production motivation [59]. The situation experienced in primary care centres during the COVID-19 pandemic did not allow the workforce to develop a moral identity [29]. This risked generating indifference, lack of commitment, and disengagement as a defence mechanism.

Zygmunt Bauman [29] suggested that feelings of uncertainty, insecurity, and lack of protection are a common experience in a new modernity defined by its fragility. Security and confidence are a characteristic of times past, while flexibility and instability represent a new world defined by constantly changing demands – with no guarantee that abilities or experiences are ever going to be enough. Indeed, the crisis caused by the SARS-CoV-2 virus has only accelerated already existing processes and dynamics, affecting the working conditions of healthcare professionals worldwide. In particular, making decisions against their professional ethical standards and witnessing the unequal distribution of healthcare resources among the population caused an added moral strain [60].

Bauman, from the viewpoint of social theory, has engaged in an intensive process of reflection on the “liquid” quality of life in modern society, exploring and applying this metaphor to personal life, time, love relationships – experienced under conditions of fluidity, instability, uncertainty, and constant adaptation. Bauman [29] defined “liquid life” as a “society in which the conditions under which its members act change faster than it takes the ways of acting to consolidate into habits and routines”. Changes experienced within a social organisation require a certain degree of continuity for people to acquire a shared perception of these new values, and shape their actions accordingly. Ledema and Scheeres [17], as part of an emerging trend of critical reflection and theorisation on healthcare team dynamics, have argued that these workers are subjected to rapid changes and high levels of ambiguity, leading to a redefinition of their professional identities. Even before the COVID-19 pandemic, healthcare teams were being put together in an ad hoc manner – inter-team collaboration being increasingly frequent, more complex, and difficult to coordinate [58] and thus requiring ever-increasing flexibility from the workers.

The “liquid” healthcare strategies described in our participants’ accounts are associated with the emergence of new working practices characterised by constant improvisation, instability, and increasing adaptability. The contributions of Engeström [61] and, in particular, Bleakley [58] to define teamwork within liquid healthcare environments have revealed a shift in priorities: instead of seeking stability in teams and networks, a new appreciation has emerged of the importance of instability – flexibility, improvisation, and “knotworking” – within healthcare teams. That is, a dynamic, negotiated, and collaborative approach to “unknotting” problems. Bleakley’s definition of knotworking suggests embracing complexity and uncertainty, capitalising on their potential as innovative resources for developing new knowledge and skills to foster adaptability in uncertain, complex scenarios. Studies on resilience, the human capacity to successfully adapt to adverse circumstances [62] share this approach and its relational aspects – with interaction and connections between people being critical for developing resilience, which is thus a collective, dynamic process [59].

These theories also underpin current trends within British healthcare policies to address society’s practical necessities and realities. They have also shaped the emergence of “liquid” specialist recruiting consultancies focused on healthcare workers [58]. Demands among Spanish healthcare professionals for a new management model must be understood from this novel viewpoint – that more “flexible” strategies might be more efficient.

On the other hand, the scenario described by our participants – lack of motivation and feeling unappreciated, wanting a more horizontal approach to decision-making – has been noted as fostering new, more flexible working management practices [16–20]. Organisational culture [13] is considered as a network or structure with shared meanings, created through a collaborative communication process between members of an organisation. An organisational culture of poor communication prevailed in a moment when clear, simple, specific messages were more necessary than ever. As a way forward for the future, we suggest healthcare authorities include primary care professionals in decision-making processes. It is also important to bear in mind that integrating a wide range of viewpoints in the planning process [63] provides a more comprehensive assessment of situations and decisions, despite its inherent difficulties.

Healthcare workers endured the lack of clear guidance during the first wave of infections by relying on their professionalism and a vocational attitude towards their work [50]. This was a factor that allowed them to cope with poor working conditions. The weaknesses appreciable in the healthcare system were dealt with through individual resourcefulness, longer working hours, re-imagining their roles, and adopting new ones.. This created further dissatisfaction among primary care workers, who felt unprotected and worried that certain attitudes –would become entrenched in the system..

The lack of engagement between workers and management teams is characteristic of our new modernity. Paying attention and looking after others, looking after public services, taking responsibility as a community for what happens within it, seem to be increasingly complicated issues. The conditions were created to start accepting certain behaviours bordering on what is morally or ethically acceptable – behaviours that become progressively irrelevant in a process of increasing adaptation, resignation, or indifference. This moral blindness is what Bauman and Donskins has termed “adiaphorisation” – a process of disengagement through which actions become neutral, irrelevant [64].

Healthcare workers’ perceived lack of support and appreciation could impact their commitment and the general quality of patient care [65]. The lack of policies and contingency plans for pandemic preparedness in the Spanish healthcare system has long been stressed, together with the chronic underfunding of primary care – whose structural weaknesses have been magnified during this health crisis. As a result, current management units – particularly regional healthcare authorities– were inadequately suited to their role in containing the spread of the pandemic.

These shortcomings reveal the deficiencies of a healthcare system that only existed as “the best in the world” in rhetorical political discourses – weighed down by material and immaterial deficiencies. At the same time, the gradual decline of the Spanish primary care system means the full extent of its fragility was never properly appreciated.

The authors consider the present pandemic provides an opportunity to capitalise on this fluid scenario of constant change and adaptation – to listen to the demands of primary care professionals to reshape the organisational model of primary care, increasing its resilience to respond to and anticipate future health crises. It is also an opportunity to reinforce and focus on policies aimed at reconstructing public services and offering strategies for healthcare leadership [66]. The institutional analysis of organisations such as the Spanish healthcare system [35] enabled us to explore its internal conflicts and contradictions, which had remained invisible before the COVID-19 pandemic. However, this health crisis has acted as a catalyst – transforming normally stable structures, while also exposing their fragility and allowing their examination.

### Limitations

The main limitation of our study is its focus on primary care settings, whose activity has been dramatically reduced during the pandemic. Primary care professionals ensured the delivery of non-COVID care despite a rise in absence rates among their workforce due to COVID-19 infections. Over the course of the study, participants shared a perception of being overburdened. On the other hand, our research project was not the only one that took place at the time. This might have caused a certain degree of saturation and reluctance to participate among primary care professionals. It is also possible that those willing to participate were particularly sensitive to deficiencies in the primary care model. Finally, the representation of different healthcare professionals has been unequal – 49% of the participants were female nurses, which might have biased the results obtained. However, this could also be a positive factor since nurses have played a crucial role in the management of primary care settings. Thus, we believe their opinions are relevant in this moment of instability and might suggest new avenues for primary care provision.

## Conclusion

The management and organization of primary care during the COVID-19 pandemic has highlighted and reinforced shortcomings that the health system already had, also in the Spanish context, considered one of the best in the world. The health crisis has highlighted structural weaknesses in the primary care model that have been magnified in the labor relations of its workers. Individualized and isolated work has been favored over teamwork. Instability and uncertainty remain beyond the first wave. Communication channels between health authorities and primary care staff do not have a horizontal approach that reflects and addresses the needs and proposals of workers based on the daily praxis of the teams. In this context, the capacity to adapt to a changing environment is valued as an innovative resource, where the flexibility of workers is the key to solving problems or dissolving complex knots.

This analysis of health policies at the micro level has identified a blurring of the professional identity of primary care workers in a context of unstable, increasingly improvisational, inconsistent, and changing labor management models and organizational strategies. The COVID-19 pandemic has highlighted the lack of key leadership skills to face extreme situations, as well as the subordinate position of the primary care model in relation to specialized care. In this sense, its workers are calling for a new primary care with the necessary resources to enable them to do their job well and regain their working dignity.

It is necessary to review the channels of communication between managers and workers to reach consensual decisions. Future studies should evaluate health policies at the macro level and improve the leadership skills of health managers.

## Data Availability

The data presented in this study are available on request from the corresponding author. The data are not publicly available due to confidentiality agreements with participants.
